# Classical Orbital Floor Post-Traumatic Reconstruction vs. Customized Reconstruction with the Support of “In-House” 3D-Printed Models: A Retrospective Study with an Analysis of Volumetric Measurement

**DOI:** 10.3390/diagnostics14121248

**Published:** 2024-06-13

**Authors:** Elvis Kallaverja, Ida Barca, Francesco Ferragina, Maria Giulia Cristofaro

**Affiliations:** 1 Department of Experimental and Clinical Medicine, Maxillofacial Surgery Unit, Renato Dulbecco Hospital, Magna Graecia University of Catanzaro, 88100 Catanzaro, Italy; reikey2003@yahoo.com (E.K.); francesco.ferragina92@gmail.com (F.F.); 2 Department of Experimental and Clinical Medicine, Maxillofacial Surgery Unit, Magna Graecia University, 88100 Catanzaro, Italy; barca.ida@gmail.com

**Keywords:** blow-out fracture, orbital floor reconstruction, patient-specific mesh, in-house 3D support, orbital volume, functional outcome

## Abstract

Background: Orbital floor fractures (OFFs) represent an interesting chapter in maxillofacial surgery, and one of the main challenges in orbit reconstruction is shaping and cutting the precise contour of the implants due to its complex anatomy. Objective: The aim of the retrospective study was to demonstrate, through pre- and postoperative volumetric measurements of the orbit, how the use of a preformed titanium mesh based on the stereolithographic model produced with 3D printers (“In-House” reconstruction) provides a better reconstruction volumetric compared to the intraoperatively shaped titanium mesh. Materials and Methods: The patients with OFF enrolled in this study were divided into two groups according to the inclusion criteria. In Group 1 (G1), patients surgically treated for OFF were divided into two subgroups: G1a, patients undergoing orbital floor reconstruction with an intraoperatively shaped mesh, and G1b, patients undergoing orbital floor reconstruction with a preoperative mesh shaped on a 3D-printed stereolithographic model. Group 2 (G2) consisted of patients treated for other traumatic pathologies (mandible fractures and middle face fractures not involving orbit). Pre- and postoperative orbital volumetric measurements were performed on both G1 and G2. The patients of both groups were subjected to the measurement of orbital volume using Osirix software (Pixmeo SARL, CH-1233 Bernex, Switzerland) on the new CT examination. Both descriptive (using central tendency indices such as mean and range) and regressive (using the Bravais–Pearson index, calculated using the GraphPad program) statistical analyses were performed on the recorded data. Results: From 1 January 2017 to 31 December 2021, of the 176 patients treated for OFF at the “Magna Graecia” University Hospital of Catanzaro 10 fulfilled the study’s inclusion criteria: 5 were assigned to G1a and 5 to G1b, with a total of 30 volumetric measurements. In G2, we included 10 patients, with a total of 20 volumetric measurements. From the volumetric measurements and statistical analysis carried out, it emerged that the average of the volumetric differences of the healthy orbits was ±0.6351 cm^3^, the standard deviation of the volumetric differences was ±0.3383, and the relationship between the treated orbit and the healthy orbit was linear; therefore, the treated orbital volumes tend to approach the healthy ones after surgical treatment. Conclusion: This study demonstrates that if the volume is restored within the range of the standardized mean, the diplopia is completely recovered already after surgery or after one month. For orbital volumes that do not fall within this range, functional recovery could occur within 6 months or be lacking. The restoration of the orbital volume using pre-modeled networks on the patient’s anatomical model, printed internally in 3D, allows for more accurate reconstructions of the orbital floor in less time, with clinical advantages also in terms of surgical timing.

## 1. Introduction

Orbital floor fractures (OFFs) represent an interesting chapter of maxillofacial traumatology that is much debated in the scientific community regarding its epidemiological, clinical, and demographic characteristics [1,2]. Their incidence is increasing, probably due to the increase in road accidents, sporting accidents, and physical assaults. The literature contains conflicting indications and recommendations regarding orbital floor reconstruction, in particular on the operating times, the surgical approach, and the materials to be implanted, because of the great variety of options available today. All authors, however, agree in establishing that a detailed clinical examination associated with a computed tomography (CT) study is fundamental for the definition of an indication and an adequate treatment. Traumatic alterations of the orbital structures can cause entrapment of the periorbital tissue with symptoms such as diplopia, dystopia, impaired ocular motility, and enophthalmos [3]. The indication for surgery should be assessed individually for each patient after careful clinical and radiographic evaluation [4,5,6]. The primary goal of surgery is to restore orbital volume by preserving both function and aesthetics [7]. The material used to restore orbital volume should be similar to the orbital bone, easy to handle, and provide a stable result throughout life [8]. Nowadays, intraoperatively conformed titanium mesh is frequently used. This involves modeling the plates and adapting them to the shape and size of the OFF [9], until reaching an ideal conformation. For this practical reason, combined with the complex three-dimensional anatomy of the orbit and limited surgical access (which limits vision during surgery), orbital reconstructions remain a significant challenge for surgeons [10,11]. Three-dimensional technology has certainly helped modern medicine and has offered the possibility to create customized anatomical models through 3D printing. In this way, it was possible to evaluate traumatic pathologies not only on a virtual screen but also on stereolithographic models. Titanium plates shaped before surgery on the stereolithographic model (produced with 3D printers) are much more precise and require less surgical time and less damage to the tissues of the patient. The future projects us toward specific patient implants, but currently, not all centers can afford such a model, especially concerning costs. In the beginning, 3D printing had high costs and long production times [10,12]; often, 3D printing was carried out at other facilities, even distant ones, and consequently delivery times were long [13]. All this led to a limited use of such prototyping in the medical field. Nowadays, the introduction of in-house 3D printers for producing orbital models is gaining increasing popularity in clinical and surgical practice [14,15,16], achieving high precision at a low cost. Through volumetric measurements of isolated and unilateral orbital floor fractures, comparing the postoperative volume with the healthy contralateral, the authors wanted to demonstrate how the use of a preformed titanium mesh based on the stereolithographic model produced with 3D printers (“In-House” reconstruction) provides a better volumetric reconstruction compared to intraoperatively conformed titanium mesh.

## 2. Materials and Methods

This retrospective study included patients surgically treated for OFF at the Maxillofacial Traumatology Center of the “Magna Grecia” University Hospital of Catanzaro, from 1 January 2017 to 31 January 2021. The study was carried out according to the guidelines set out in the Helsinki Declaration and was approved by the Ethics Committee of the “Magna Grecia” University of Catanzaro, Italy. The aim of this study was to compare two titanium mesh modeling techniques using a volumetric analysis of the orbits in patients subjected to surgery for OFF.

The inclusion criteria were as follows:

- A diagnosis of unilateral isolated OFF in male patients aged >18 years;
- Pre- and postoperative ophthalmological examination with preoperative diplopia confirmed by an Orthoptic examination with Hess Lancaster screens;
- Pre- and postoperative CT in our Radiology Center with slice of 0.5 mm;
- An absence of muscle deficits and nerve or vascular damage;
- Surgery performed within 2 weeks from trauma;
- Three-dimensional stereolithographic printed model, volumetric measurements, and reconstruction with titanium mesh (both modeled intraoperatively and preoperatively on a 3D model) performed by the same surgeon.

The exclusion criteria were as follows:

- Patients with multiple maxilla-facial fractures;
- Prior surgery for orbital trauma or ophthalmic surgery;
- Patients who were not cooperating;
- Patients with incomplete clinical-radiological documentation;
- A reconstruction of the orbital floor with other materials.

### 2.1. Procedure

All patients arrived, hemodynamically stable, from the Emergency Departments of other local hospitals with radiological exams and urgent specialist visits. After clinical and radiological consultation, patients were hospitalized in case of surgical indication. The study design and treatment protocol are shown in Table 1.

Each patient underwent a general and loco-regional objective examination (including weight, height, and vital signs), instrumental examinations such as thin layer CT (0.5 mm) with axial, coronal, and sagittal acquisitions and 3D reconstructions, ophthalmological examination pre- and postoperatively, and orthoptic examination with Hess-Lancaster screens. We also classified the blow-out fractures according to Harris’ classification. Supported by the literature, indications for surgical reconstruction were fractures with defects ≥ 3 cm^2^, enophthalmos ≥ 2 mm, dislocated fractures, and soft tissue herniation [5,6]. 

Patients enrolled in the study were divided into two groups:

Group 1 (G1): patients surgically treated for isolated OFF, divided into 2 subgroups: G1a (patients undergoing orbital floor reconstruction with an intraoperatively shaped mesh) and G1b (patients undergoing orbital floor reconstruction with a preoperative mesh shaped on a 3D-printed stereolithographic model, obtained from the patient’s CT scan and using the Ultimaker S5 3D printer—Manufat Engineering Srl, 160 23900 Lecco (LC), Italy).
Group 2 (G2): patients treated for other traumatic pathologies such as mandibular or middle third fracture not involving orbit (zygmatic arch, Le Fort I, etc.).

Pre- and postoperative orbital volumetric measurements were performed on both G1 and G2.

Patients of both groups were subjected to the following:

- Measurement of orbital volume using Osirix software (Version 12.0) (Pixmeo SARL, CH-1233 Bernex, Switzerland) on the new CT examination. This was carried out both semi-automatically and manually. For each patient, the orbital volume on the preoperative CT was calculated for both healthy and fractured orbits. On the postoperative CT, however, the orbital volume reconstructed with titanium mesh was calculated. We then calculated the difference in volumes between healthy and traumatized orbit, both before and after surgery. Finally, we evaluated the recovery difference, which is made up of the difference between the two volumes, pre-op and post-op, of the traumatized orbit.
- Post-surgery thin layer CT (0.5 mm) with axial, coronal, and sagittal acquisitions and 3D reconstructions.

On this printed model we modeled, before surgery, the titanium mesh. All preoperative stages (study and printing), surgery, and postoperative studies were performed by the same surgeon, to reduce bias.

### 2.2. Measurement of Orbital Volumes

**Figure 1:**
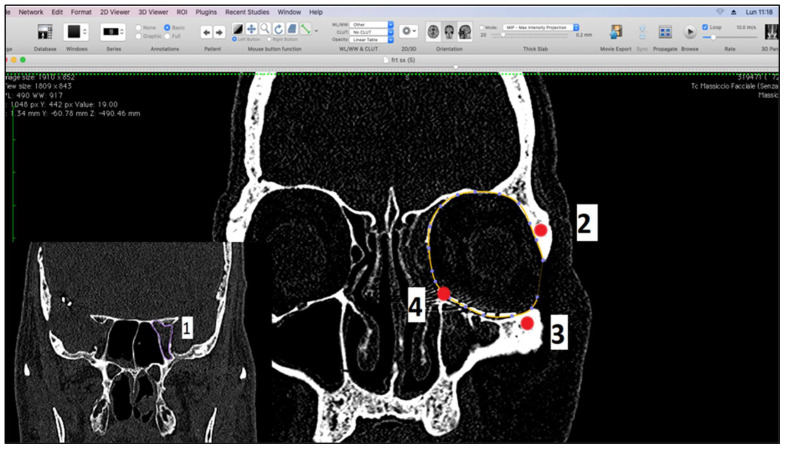
Measurement landmarks.

We performed the measurements using Osirix software in semi-automatic and manual mode on CT scans [17,18], on both preoperative and postoperative CT examinations, with slices of 0.5 mm and with the head positioned in its “neutral position”. Preoperative CT was carried out within 24 h of the trauma. We corrected the minimal movement discrepancies using the MPR function of the software with anatomical references in the planes: the Frankfurt horizontal plane, a vertical plane passing between the supraorbital foramen and the maxilla-malar suture of the lower orbital frame, and the tangential horizontal plane to the lower orbital frames. Volumetric analysis after CT orientation was performed on coronal scans in bone cuts. Since the orbital cavity reflects the geographical shape of a cone, with the base at the level of the orbital frames, several reference points have been identified to standardize the measurement procedure which are (1) the optic foramen; (2) the frontal zygomatic suture; (3) the maxillo-malar suture at the level of the lower orbital frame; and (4) the lacrimal canal. The plane passing through points 2, 3, and 4 was considered the base of the cone (Figure 1).

**Figure 2:**
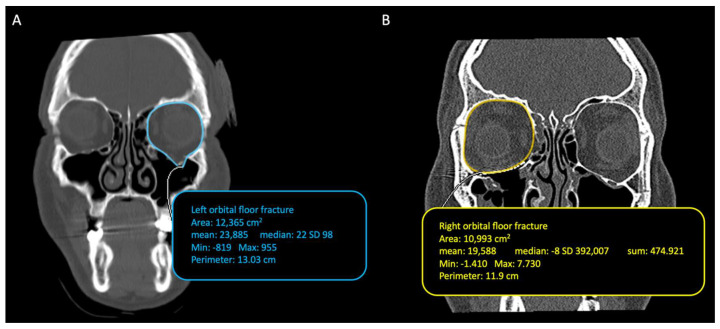
(**A**) Preoperative orbital volume measurement including herniated soft tissues. (**B**) Postoperative orbital volume measurement.

Using the option ROI (region of interest) and selecting the tool “closed polygon”, present on the software Osirix, we could manually identify and delineate the regions of interest for each section of the cut. This was done using the reference points in the posterior to the anterior direction (from the apex to the base of the orbit). Figure 2A shows the preoperative orbital volume measurement including herniated soft tissues. Instead, Figure 2B shows the measurement of the postoperative orbital volume; we performed the measurements following the neo-floor (following the titanium mesh).

**Figure 3:**
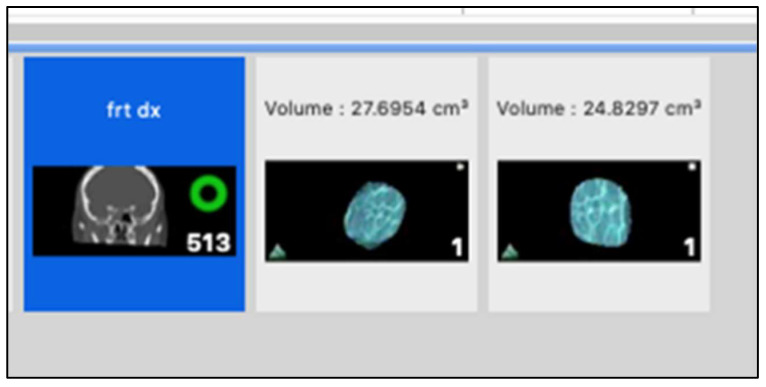
Volume calculation.

After contouring each slice of the CT scan, we applied the volume calculation function of the selected ROI (Figure 3). We reported all calculated data in an Excel (Version Microsoft 365) table on which the statistical analysis was carried out.

### 2.3. Production of the 3D Model

**Figure 4:**
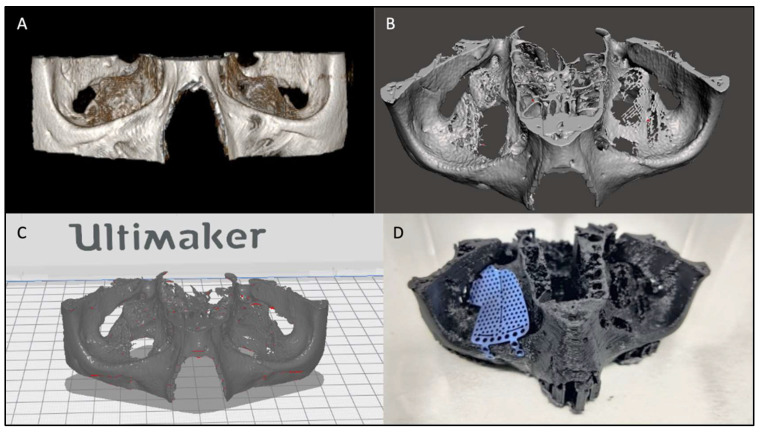
(**A**) DICOM file of a 3D reconstruction, using Radiant. (**B**) Meshmixer solid stereolithographic file. (**C**) Ultimaker CURA 3D printing file. (**D**) Titanium mesh shaped on the 3D printed patient anatomical model.

After acquiring the preoperative CT (performed on 0.5 mm bone cuts), we exported the DICOM file using the Radiant software and created the 3D model with the acquisition of the post-processing stereolithographic file. So, we created an stl. file with the bone part of interest (Figure 4A). The stereolithographic file was opened with another open-source software called Meshmixer (Version 3.5) (Figure 4B). In this software, the processing and cleaning of the model were practiced creating a solid file that could be printed on 3D printers. Finally, the stereolithographic file was linked to the CURA software (version 4.2) specific to the Ultimaker (Zaltbommel, The Netherlands) 3D printer used for this study (Figure 4C). The stereolithographic model generated by 3D printing was inserted and modeled on the fractured orbit of the patient (Figure 4D).

The modeling criteria were based on basic principles, such as fixed points of the residual orbital floor where the mesh can be placed to have a support base; restoring the anatomy of the floor as faithfully as possible by comparing it with the contralateral floor; and retaining important anatomical structures that may be damaged during placement, such as the lower orbital fissure, the lacrimal sac, and the infraorbital nerve.

### 2.4. Statistical Analysis

We performed both descriptive and regressive statistical analyses on the recorded data.

We performed descriptive statistical analysis using central tendency indices (such as mean and range) and absolute and relative frequencies for categorical data. A descriptive analysis was performed for an overview of the data. The averages of the orbital volumes give us an idea of the central value of the data, while the standard deviations give us a measure of how dispersed the data were around the mean.

We performed regressive statistical analysis using the Bravais–Pearson index, calculated using the GraphPad program (Version 10.2.3) (GraphPad Company, San Diego, CA, USA). This was used to evaluate the relationship between the healthy orbital volume and the surgically treated orbital volume in both groups.

## 3. Results

In the timeframe analyzed from 1 January 2017 to 31 December 2021, we evaluated 176 patients treated for OFF and 10 fulfilled the study’s inclusion criteria. The sample examined included patients with an average age of 39.5 years and a range of 16–88 years. The most frequent cause of trauma was road accidents (n. 4, 40%), followed by domestic accidents (n. 3, 30%), sports injuries (n. 2, 20%), and interpersonal violence (n. 1, 10%). 

The 10 patients of G1 were divided into two groups, with 5 assigned to G1a and 5 to G1b, and a total of 30 volumetric measurements were performed, 20 preoperatively and 10 postoperatively.

In G2, we included 10 patients treated for other traumatic pathologies (face lower third fractures), and a total of 20 measurements were performed.

The average volume based on the literature is about 24.5 ± 3.08 cm^3^ [19]. Compared to these data, ours remains an effective and reliable measurement method with a volumetric mean of healthy orbits of 24.511 cm^3^, with a standard deviation of 2.30. Instead, the mean volume of fractured orbits was 27.318 cm^3^, with a standard deviation of 2.87. After treatment, the orbits reconstructed with the mesh had a volumetric mean of 24,089 cm^3^, with a standard deviation of 2.674. The maximum herniation was approximately 4.4098 cm^3^, and the minimum herniation was approximately 1.0433 cm^3^. 

Table 2 and Table 3 present the data of patients treated with a conventional intra-op-shaped titanium mesh and with a titanium mesh pre-op-shaped based on the 3D printed model, respectively. 

We saw that in four out of five patients the volume of the traumatized orbit, after surgery, was greater than the healthy contralateral; the recovery difference had a negative value. In one case, it was smaller, and the recovery difference had a positive value. The range of recovery difference was from −1.9963 to +1.5689 cm^3^. These patients had a resolution of diplopia within 2–6 months, as evaluated with the Hess Lancaster test. The average surgical timing was 79 min. In two cases, the patient suffered from postoperative exophthalmos due to hypercorrection. No patient had dystopia.

The descriptive analysis of G2 in Table 4, demonstrated that the mean of the volumetric differences was ±0.6351 cm^3^ and the standard deviation of the volumetric differences was ±0.3383. These data allowed us to evaluate and compare how much the mean and standard deviation of the volumetric differences approached or moved away from G1 patients compared to G2.

We saw that in three out of five patients, the volume of the traumatized orbit, after surgery, was greater than the healthy contralateral. In two cases, it was smaller. The range of recovery difference was from −0.6767 to +0.4681 cm^3^. These patients had a resolution of diplopia in less than 1 month, as evaluated with the Hess Lancaster test. The average surgical timing was 39 min. No patient had dystopia, exophthalmos, or enophthalmos.

**Figure 5:**
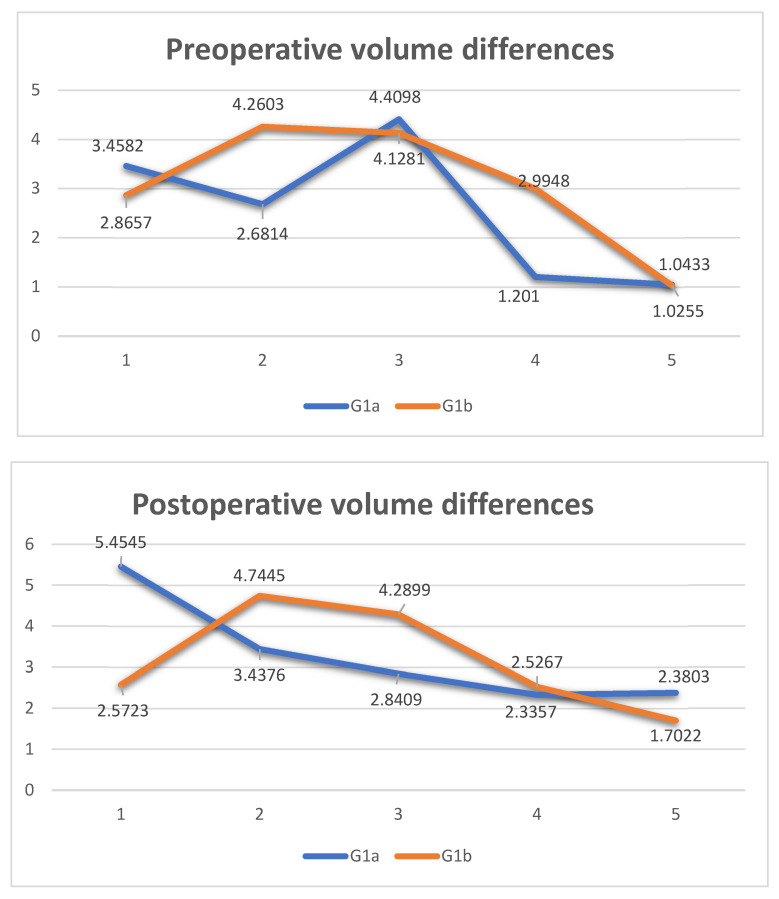
Comparison of the differences in preoperative and postoperative volumes.

Figure 5 shows two graphs comparing the differences in the volumes of patients in groups G1a and G1b, preoperatively and postoperatively. A very important result, despite the limited number of patients, is the trend of the two orange and blue lines, respectively, of groups G1a and G1b. In both charts, the blue line has a much more uniform pattern than the orange one. Therefore, we can say that in G1b, the volume recovery from the preoperative to the postoperative period was more linear than in G1a.

**Figure 6:**
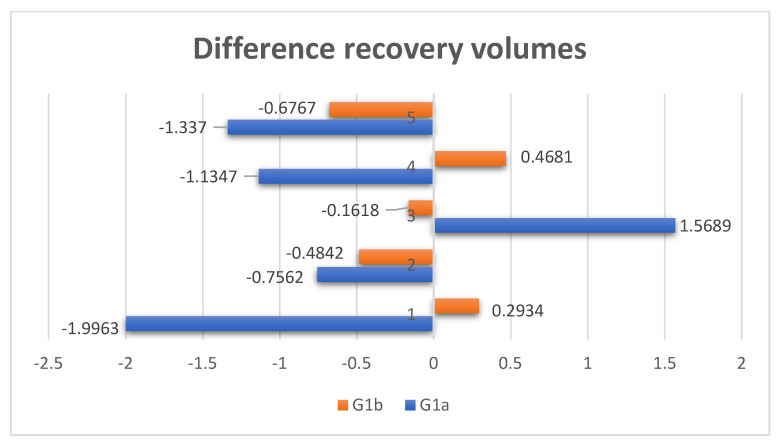
Comparison of the difference in recovery volumes.

Figure 6 shows the recovery volume in the two groups. This difference should ideally be 0. The closer the result is to 0, the more likely it is that the surgically treated orbit will have an optimal recovery. This is well highlighted in group G1b.

**Figure 7:**
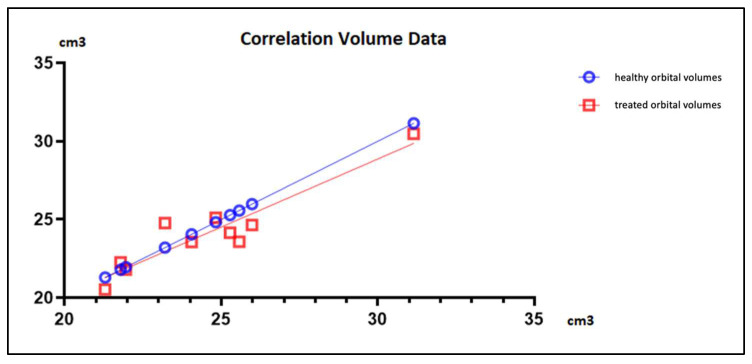
Graph showing the linear relationship between healthy orbital volumes and treated orbital volumes.

As a final analysis, we calculated the Bravais–Pearson index. It emerged that the relationship between healthy orbital volumes and treated orbital volumes was linear (Figure 7), and both variables were distributed according to a normal one. Significance was confirmed by a value *p* < 0.0001, with an r value = 0.9350. The 95% confidence interval was between 0.7425 and 0.9849.

**Figure 8:**
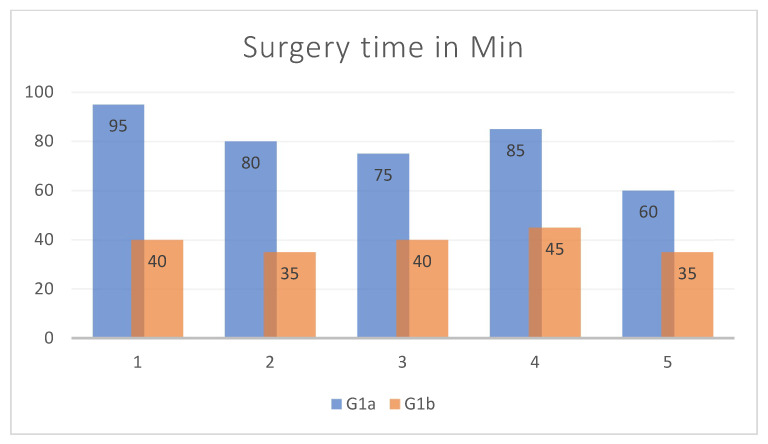
Comparison of surgical timing between G1a an G1b.

From the analysis of the surgical times used, it appears that G1b had an average surgical timing of about 40 min less than G1a (Figure 8).

## 4. Discussion

At present, there is no doubt that the presence of diplopia represents a surgical indication in patients with OFF, but even today, the debate remains open in the literature regarding the best way to perform the operation and the type of material to be implanted. The advantages and disadvantages of the most used materials for reconstruction as well as the methods of their use have been widely described in the literature [20,21,22]. Titanium is an excellent material for implants due to its properties such as high biocompatibility, less-than-one-millimetre thickness, and excellent malleability and stability. The increased precision of titanium implants improves the fit of the implant and minimizes the number of insertion tests, causing minor damage to the surrounding soft tissues [23,24,25]. Titanium plates modeled before surgery on the stereolithographic model (produced with 3D printers) are much more precise and require less surgical time and less damage to the patient’s tissues. In the beginning, 3D printing had high costs and long production times [10,26]; often, 3D printing was carried out at other facilities, even distant ones, and consequently delivery times were long [27]. All this led to a limited use of such prototyping in the medical field. In our study, the models were made using an in-house 3D printer, and this was certainly less expensive and took less time than the external printing of standard stereolithographic models [25]. The rigorous inclusion criteria adopted allowed us to minimize selection bias and to make the sample even more homogeneous, as we considered only male patients. Similarly, including asymptomatic patients immediately after surgical treatment did not influence the functional results. This allowed us to choose patients with great precision for about 5 years. From the analyses carried out and the results obtained, we can say that the measurements are quite reliable. From these results, we can deduce that a post-treatment volumetric recovery of the fractured orbit will be closer to a healthy orbit with less probability of having distant diplopia. Observing the differences in the standard deviation between G1a and G1b, it was seen that in the five patients belonging to G1a, the standard deviation moved away from that measured in group G1b (±0.338), with an excess or deficit volumetric recovery. In four of these patients, the residual diplopia resolved within 6 months. In one patient (standard deviation difference of −1.9963), diplopia persisted one year later. Therefore, although the volume restored by surgery is not exactly the pre-trauma volume, it is included within a standardized range of orbital volume. All this translates into better aesthetic and functional results and therefore less likelihood of diplopia persisting after surgery. The study by Holmes and Schlittler [28] showed a statistically significant improvement through the use of patient-specific implants, especially in larger and more complex fractures. Zimmerer et al. [29] showed no significant differences in postoperative diplopia and ocular motility between the two groups examined. Instead, Kozakiewicz et al. [30] showed that ophthalmic results were better in the long term for individual orbital systems. Kim et al. [31] showed a higher rate of postoperative resolution or the persistence of only mild symptoms in the group with the patient-specific pre-shaped implants, while the conventional group also experienced moderate to severe postoperative symptoms. [29,32,33,34]. According to previous studies, the reduction in surgical timing in G1b showed a significant statistically significant difference, with an average recovery of 40 min, less intraoperative stress, and a faster postoperative recovery [35,36]. To improve the result and comparison in the study, we preferred to perform measurements of orbital volume. The literature does not report a gold standard method for measuring orbital volume. We chose to use medical software like Osirix MD that allows measurements to be performed on CT scans. The models were made using an in-house 3D printer, and this made it less expensive and time-consuming than externally printed standard stereolithographic models [25]. The virtual design, 3D printing, and preoperative bending of the titanium mesh took 50 to 120 min, without causing delays in surgical timing. The accuracy of low-cost 3D printed models was found to be satisfactory for daily maxillofacial procedures, such as teaching purposes, preoperative surgical planning, and plate bending [14,15,32,36].

## 5. Conclusions

“In-house” pre-modelling on stereolithographic models, compared to that practiced intraoperatively, has a significantly higher accuracy in restoring the fractured floor as faithfully as possible and thus also restoring orbital volume. This study demonstrated that in patients with a pre-modeled mesh, the orbital volume fell within the target range of the standard deviation, with diplopia disappearing immediately postoperatively or after 1 month. This result demonstrates that this modeling method allows for greater precision in the contours and a less invasive insertion of the implant, avoiding phenomena of multiple attempts at adaptation or malpositioning. Our results in the orbital volumetric analysis suggest the use of this method which involves better volumetric recovery, greater precision, better aesthetic results, earlier functional results, less surgical time, and better post-op recovery. Therefore, ours remains a valid and reproducible type of surgical treatment.

## Figures and Tables

**Table 1:** Treatment protocol.

10 patients isolated blow out fracture	
CT scans and Hess Lancaster screen	
**G1a** (intraoperative shaping mesh)	**G1b** (preoperative shaping mesh on 3D printed stereolithographic model)
	Import Dicom blow out fracture Into Osirix MD software
	Blow Out fracture identification, first volume measurements, export the Dicom file in the stl file
	3D Printing of the stereolithographic model
	Preoperative shaping of the mesh
	Sterilization of the pre shaped mesh
	Titanium mesh insertion without further adjustment
	Postoperative CT
	Import the new Dicom into Osirix MD software
	Second volume measurement
	Creating Table 2 and Table 3
Intraoperative shaping, mesh insertion, and evaluation if shape and insertion are correct	Analysis

**Table 2:** Data of patients in G1a (treated with intra-op-shaped titanium mesh).

	Age	Sex	Type of Incidente	Fracture Side	Volume cm^3^ Right Orbit	Volume cm^3^ Left Orbit	Volume cm^3^ Mesh Orbit	Vol- cm^3^ Difference Pre-op	Vol- cm^3^ Difference Post-op	Vol- cm^3^ Recovery Difference	Surgery Time in Min
**1**	33	Male	aggression	Right	29.0359	25.5777	23.5814	3.4582	5.4545	−1.9963	95
**2**	59	Male	aggression	Right	23.9741	21.2927	20.5365	2.6814	3.4376	−0.7562	80
**3**	37	Male	car accident	Left	23.204	27.6138	24.7729	4.4098	2.8409	1.5689	75
**4**	74	Male	accidental fall	Left	25.2797	26.4807	24.145	1.201	2.3357	−1.1347	85
**5**	41	Male	aggression	Left	25.9815	27.0248	24.6445	1.0433	2.3803	−1.337	60

**Table 3:** Data of patients in G1b (treated with preop-shaped titanium mesh).

	Age	Sex	Type of Incidente	Fracture Side	Volume cm^3^ Right Orbit	Volume cm^3^ Left Orbit	Volume cm^3^ Mesh Orbit	Vol- cm^3^ Difference Pre-op	Vol- cm^3^ Difference Post-op	Vol- cm^3^ Recovery Difference	Surgery Time in Min
**1**	19	Male	sports trauma	Right	27.6954	24.8297	25.1231	2.8657	2.5723	0.2934	40
**2**	72	Male	car accident	Left	24.0521	28.3124	23.5679	4.2603	4.7445	−0.4842	35
**3**	37	Male	aggression	Right	21.9546	26.0827	21.7928	4.1281	4.2899	−0.1618	40
**4**	30	Male	sports trauma	Left	21.7904	24.7852	22.2585	2.9948	2.5267	0.4681	45
**5**	19	Male	sports trauma	Left	31.149	32.1745	30.4723	1.0255	1.7022	−0.6767	35

**Table 4:** Data of patients in G2.

	Age	Sex	Volume cm^3^ Right Orbit	Volume cm^3^ Left Orbit	Volume cm^3^ Difference
**1**	23	Male	24.3835	25.5133	1.1298
**2**	55	Male	24.2495	24.7679	0.5184
**3**	39	Male	24.4433	24.5261	0.0828
**4**	47	Male	20.2659	20.5763	0.0828
**5**	53	Male	23.5792	22.8334	0.7458
**6**	28	Male	22.7471	23.3860	0.6389
**7**	19	Male	27.9404	27.2011	0.7393
**8**	38	Male	23.0517	21.9158	1.1359
**9**	46	Male	23.5677	24.1943	0.6266
**10**	57	Male	28.6717	29.1944	0.5227

## Data Availability

The data presented in this study are available on request from the corresponding author.
